# Clonal fidelity investigation of micropropagated hardened plants of jackfruit tree (*Artocarpus heterophyllus* L.) with RAPD markers

**DOI:** 10.1186/s43141-022-00426-0

**Published:** 2022-10-20

**Authors:** Abdul Kader, Sankar Narayan Sinha, Parthadeb Ghosh

**Affiliations:** Department of Botany, University of Kalyani, Kalyani, Nadia, West Bengal India

**Keywords:** Acclimatization, Agroforestry, Establishment of culture, Genetic fidelity, Genetic uniformity, Importance of jackfruit, In vitro rooting, Media screening, Somaclonal variation, Subculture

## Abstract

**Background:**

*Artocarpus heterophyllus* is an important tropical agroforestry species that bears multiple applications. However, the population of this species is reduced due to various anthropogenic activities. For this reason, in vitro approach is needed to propagate or conserve this species as in vivo propagation methods face various obstacles. In this respect, the present investigation was undertaken to produce genetically stable jackfruit trees through in vitro technology. In vivo grew shoot tips were harvested on Murashige and Skoog (MS) medium containing several plant growth regulators to achieve this.

**Results:**

The 6-benzylaminopurine (BAP) at the concentration of 1.5 mg L^-1^, indole-3-butyric acid (IBA) at 0.5 mg L^-1^, and α-naphthaleneacetic acid (NAA) at 0.1 mg L^-1^ in combination on MS media yielded superior shoot response (94.44%), longest shoot length (4.02 cm), and the maximum number of shoots per explant (4.78). They were further multiplied by repeated subculturing on the same media composition and the third subculture resulted in a maximum number of shoots (5.92) with the largest shoot length (5.85 cm). Among the different media screened for rooting, the ¼ MS media yielded 94.44% rooting response, the longest root length (3.78 cm), and the maximum number of roots per shoot (8.44) with 0.1 mg L^-1^ NAA, 0.5 mg L^-1^ IBA and 0.1 mg L^-1^ BAP in combination. Primary hardening showed 88.89% of plant survival under greenhouse conditions after 4 weeks of incubation having a sterilized mixture of garden soil and vermiculite mixture (1:1, w/w). It increased to 90.60% after the secondary hardening process in a vermiculite-soil mixture (2:1; w/w). No polymorphism was detected on random amplification of polymorphic DNA (RAPD) profiling between the mother plant and hardened plants, indicating high genetic stability among the clones.

**Conclusions:**

This is the first report of the genetic fidelity study of in vitro grown regenerants of *A. heterophyllus*. This study established a micropropagation protocol for genetically uniform in vitro regeneration of this species to supply plant resources to various industries or conservation of elite germplasm.

## Background

The jackfruit tree (*Artocarpus heterophyllus* L.) is locally known as “kanthal” in Bengali and belongs to the mulberry family, Moraceae. This species is believed to have its origin in the Malayan rain forests and Western Ghats of India [27, 37]. This woody, monoecious, fast-growing, large, evergreen tree is developed over time and proved to be one of the most broadly distributed tree species in many tropical and subtropical countries of the world [37]. As the time spent away, it has adapted and appreciated a wider delivery in many humid tropics such as Indonesia, China, Thailand, Sri Lanka, Philippines, and portions of Africa, Costa Rica, Brazil, the Caribbean islands, Suriname, Australia, and Florida [19, 37]. In Asia, the family Moraceae has 60 genera and contains 1400 species [30]. Jackfruit, perhaps the most far and wide-circulated tree species in the genus *Artocarpus*, enjoys a leading position in tropical agroforestry predominantly on account of its multiple applications and willingness to fit in with other crop forms [27, 37]. This species is valued for its heavy yields of nutritious fruits and durable wood [2]. The jackfruit tree is easily identified from other trees based on its unique fruit which is the largest edible tree-born fruit (Getting up to 50 kg in weight and 90 cm in length) among the cultivated plants [25, 37]. It provides higher production of fruits than any other tree species in the world [2]. The jackfruit tree is eco-friendly and produces pesticide-free fruit as the tree has no serious pests and diseases [18]. A mature tree produces up to 700 fruits (0.5–50 kg weight) per year (during summer) when staple food grains are little delivered [3, 25]. The low-cost price and availability make the jackfruit to be the “poor man’s food” during the summer when the food supply is inadequate [29, 42, 50]. Due to the large size, six to eight members of a family can easily satisfy their hunger by eating one jackfruit at a time [8]. Jackfruit often takes a position in the regular diet in many places, and it is consumed by each class of people [24]. Jackfruit is rich in calories (84%), carbohydrates, proteins, vitamin A (540 IU), B and C complex, several minerals (iron, calcium, and phosphorus), and high amounts of protein, thiamin, riboflavin, calcium, and carotene [25, 33, 37, 42]. Jackfruit is broadly applied for preparing jams, jellies, chips, nectar, desserts, baby foods, biscuits, bread, and beverages like squash and wine [18]. Moreover, raw jackfruit is recommended for diabetic patients, owing to its antidiabetic assets. Having the presence of a low glucose level and fibrous nature, jackfruit is extensively acknowledged by diabetologists as a correct food source for diabetic patients [18]. There are many as 100–500 seeds (2 to 4 cm long) per single fruit, representing 8–15% of the total fruit weight. Normally, jackfruit seeds are viviparous in nature. These seeds are consumed in a roasted, boiled, or steamed manner or taken as a snack. Additionally, the seed flour is applied in the preparation of biscuits, sweets, and bread [12]. Apart from these, the seed contains Jacalin (a lectin protein) which is representative of more than 50% of the proteins of the jackfruit crude seed extract and bears various biological importance [30]. Jacalin effectively binds to human IgA, and different types of monosaccharides like d-galactose and T-antigen [18, 31]. In this respect, Jacalin has a great demand for immunological research purposes. Besides this, it has numerous health assistances like antioxidant, anticancer, and anti-aging properties [18]. The ripe fruit could be eaten either fresh or preserved in syrup whereas the unripe fruit is consumed as a vegetable. For these backgrounds, the jackfruit is announced as the state fruit of Kerala state (India) in March 2018 [18]. Furthermore, the jackfruit enjoys the crown as the national fruit of Bangladesh [25]. Besides the pleasant jackfruit, it is essentially cultivated for timber and fodder production also. It produces excellent timber with better durability and structural assets [46]. The wood of this species is applied for the production of durable strong furniture, fuel, fodder, etc. The jackfruit tree along with its fruit is also globally used in different Yunani and Ayurvedic medicinal preparations. The roots are applied to treat diarrhea, and the leaves are allowed for skin diseases and as an antidote to snake bites. Furthermore, the literature survey found that various preparations of different parts of this plant were rich in phenolic compounds, aryl benzofurans, stilbenoids, and flavonoids which displayed a large medicinal activity like antimicrobial, antineoplastic, antioxidant, anti-inflammatory, and hypoglycemic effects [12]. Apart from these, the leaf and young shoots of this species are one of the best fodders for domestic animals, especially goats. In connection with this, the freshly grown shoots along with leaves are sold in markets of West Bengal state as well as other states of India, Bangladesh, etc. Apart from these several medicinal and socioeconomic values, this tree contributes numerous functions to preserve the environment. This species serves as an important component for the sustainable development of soil through the prevention of soil erosion and the betterment of soil [42]. For these multiple usages of the Jackfruit tree, this species is nominated as deserving of priority attention by the International Centre for Underutilized Crops (ICUC) and the Commonwealth Science Council (CSC) to develop research plans for promoting the conservation, processing, and cultivation of this species [35]. However, indiscriminate and unscientifically cutting of this tree is noticed in a large number for timber production which in turn will reduce its population [18]. In this circumstance, it is very essential to conserve the jack trees for future use and the maintenance of biodiversity.

The jackfruit is a highly cross-pollinated tree. Therefore, maintenance of the genetic integrity of propagated plants is difficult through seeds as well as sexual propagules [5, 25]. For this reason propagation of the jackfruit plant from seeds is not widely accepted [8]. Moreover, germination of the tree from seeds is very much harder after a little time of storage of seeds as fresh seeds have a short shelf-life [2, 18, 33]. For their cross-pollination nature, high variation (heterozygosis) is noticed among the species of seedling origin. Apart from this, plantlets from seeds acquire a longer rotation time to achieve a fruit-bearing period than those trees circulated by vegetative approaches. Furthermore, trees germinated via seeds raise needlessly taller than those propagated via the vegetative process, which has constrained in managing and harvesting process. The conventional vegetative propagation methods are generally difficult, uneconomic, time-consuming, seasonal dependent, and mostly unsuccessful [8, 25]. The trees that have grown via vegetative propagation display dwarfism and are likely to produce branches at small ages which resulted in low-quality timber with a small trunk. In this way, this technique has drawbacks for the efficient and commercial level of propagation of this species. In this context, the tissue culture technique is the most appropriate, suitable, and better alternative to various in vivo methods to produce true-to-type plantlets all over the year in a short period. Generally, woody tree species are known to be recalcitrant in nature for micropropagation because it causes explant browning due to the secretion of phenolic compounds, takes longer periods to complete their establishment, has low in vitro response, etc. [41]. However, several reports are available on the micropropagation of *A. heterophyllus* using higher concentrations of different hormones, lacking clonal fidelity study [2, 5, 33]. The chance of somaclonal variation (A phenomenon that occurred during in vitro study) in woody tree species is much higher than in other plant species because it can take a long rotation time [54, 68]. The somaclonal variation depends on the medium used, different hormone concentrations, and culture conditions [32]. The basic principle of tissue culture is the production of true-to-type plants. So, it is vital to certify clonal fidelity for long-term economic settlement. For the detection of somaclonal variation, many methods (morphological and physiological treatment, biochemical, and karyotyping) exist, but most of these have their limits. The PCR-based molecular marker technology such as RAPD (random amplification of polymorphic DNA) is advantageous in many ways like independency of environmental factors, requires a little amount of DNA without involving a radioactivity test, easy to perform, fast, reliable, cheapest, and able to detect genetic variation in closely related species, such as two near-isogenic lines [14, 68].

Keeping these constraints in thoughts, the present study was formulated for the clonal fidelity investigation of in vitro grown plants through molecular markers like RAPD for establishing a particular micropropagation system for the production of genetically identical and stable plants before it is released for commercial purposes.

## Methods

### Plant material and explant preparation

The shoot tips were collected from healthy and juvenile shoots of a mature fruit-bearing jackfruit tree (approximately 33 years old) grown in the experimental field of the department of botany, University of Kalyani. The shoot tips were excised from branches and placed in water to bring into the laboratory. The explants were washed under running tap water, then dipped in 0.5% Bavistin (BASF India Ltd., Thane Maharastra, India) solution for 5 min and cleaned three times with double distilled sterile water. The explants were then shifted in an autoclavable jar with 0.1% HgCl_2_ solution (Himedia, Kolkata, West Bengal, India) for 8 min and washed 3 times with sterile distilled water under laminar airflow.

### Culture media and conditions for in vitro shooting and rooting experiments

The components or chemicals for media and plant growth regulators used in the tissue culture study were procured from Himedia. After sterilization, the shoot tips were cut into 1.2 cm long and incorporated into a sterilized Murashige and Skoog (MS) medium [44] having pH 5.7, sucrose 3%, and agar 0.8%. The culture medium containing the explant was incubated at 25 ± 2 °C under white fluorescent light (80 μmol m^-2^ s^-1^; Philips, India) following 16-h light and 8-h dark cycles with 60 to 65% of relative humidity.

### Shoot induction and multiplication

For shoot induction, the explants were cultured on full-strength MS medium supplemented with various concentrations of cytokinins like 6-benzylaminopurine (BAP) and 6-furfuryl-amino purine (Kn) at the concentration of 0.5 to 2 mg L^-1^ and auxins like Indole-3-butyric acid (IBA) and α-naphthaleneacetic acid (NAA) at the concentration of 0.1 to 0.5 mg L^-1^ were tested singly or in combination.

### Induction of root of micropropagated shoots

For rooting experiments, regenerated shoots from in vitro grown explants were excised and transferred to full strength, ½- and ¼-strength MS medium supplemented with different concentrations of auxins (IBA, NAA) like 0.1 to 0.5 mg L^-1^ and cytokinins (BAP, Kn) at the concentrations of 0.05 to 0.1 mg L^-1^. The results of shooting and rooting experiments were noted after 45 days of incubation. The subculturing was done after 30 days of intervals on the same media and compositions.

### Acclimatization and field establishment

After shooting and rooting experiments, the rooted small plantlets were taken out, washed to remove agar, and acclimatized initially to the ex vitro controlled environment (primary hardening). For this procedure, they were introduced into plastic pots (7.5-cm diameter) with sterilized garden soil and vermiculite mixture (1:1) in a restricted environment chamber at 25 ± 2 °C under 16-h/8-h photoperiod and relative humidity of 75 to 85%. Plants were watered with 1/8 MS basal salt solution with a hand sprayer without sucrose and inositol on alternate every three days for 2 weeks. After 2 weeks, the potted plantlets were placed into a greenhouse under 50% shade provided by Agro shade net (B&V Agro Irrigation Co., India). In the greenhouse, they provided a 10-h photoperiod light intensity of 200 μmol m^-2^ s^-1^, relative humidity of 70%, and 28 ± 2 °C–25 ± 2 °C day/night temperature. Three comparable experiments were carried out for primary hardening each with thirty-three plantlets, and the percentage of survival was recorded after 3 weeks.

In the secondary hardening process, the primarily hardened plants were further transplanted in earthen pots in a vermiculite-soil mixture (2:1; w/w) in the field conditions. In the experimental field, they provided a 30 to 40% of relative humidity, about 12 h day^-1^ photoperiod, 300–400 μmol m^-2^ s^-1^ irradiance (shaded sunlight), and 35 ± 2 °C/30 ± 2 °C day/night temperature. Each secondary hardening experiment was repeated three times with thirty-nine replicates each, and the percentage of survival was observed after 4 weeks of transferring into the field.

### Molecular analysis

The genetic stability of the micropropagated hardened plants was assessed with PCR-based RAPD analysis. For this experiment, the mother plant as well as randomly selected eleven in vitro-raised hardened plants were evaluated.

### Extraction of DNA

The genetic fidelity of micropropagated plantlets was carried out using PCR-based molecular markers like RAPD. Total DNA extraction was performed from leaves (100 mg) of micropropagated hardened plants and their donor mother plant according to the guidelines of Ghosh et al. [22]. The characterization of isolated DNA was conducted with a UV-VIS spectrophotometer (UV-3600i, Shimadzu Corporation, Kyoto Japan) and electrophoresis on 0.8% agarose gel.

### Primer selection and reproducibility

Initially 27 (10-mer) arbitrary decamer RAPD primers were randomly approved for screening of the PCR standardization which was procured from Bangalore Genei Pvt. Ltd., Bangalore, India. The reproducibility of the PCR products was evaluated using selected primers with different DNA samples isolated at different times independently from the mother and hardened plants which were amplified at different times. The comprehensive description of the RAPD primers used in this study was shown in Table 1.

**Table 1:** List of random amplification of polymorphic DNA (RAPD) primers, their sequence motifs, total number, and size of the amplified loci used for determination of genetic integrity of hardened and mother plants of *Artocarpus heterophyllus* L*.*

Sl no	Primer code	Primer sequence (5’-3’)	Number of amplified loci	Total number of bands amplified	Number of polymorphic band	Range of amplification (bp)
1	OPS-01	AAATCGGAGC	2	24	0	750–1500
2	0PS-02	GGTCCCTGAC	3	36	0	300–1200
3	OPS-03	GTCCTACTCG	2	24	0	800–1480
4	OPS-04	TGCGCGATCG	3	36	0	400–1500
5	OPS-05	AGTCAGCCAC	4	48	0	300–2000
6	OPS-06	AATCGGGCTG	2	24	0	700–1500
7	OPS-07	AACGTACGCG	4	48	0	300–2000
8	OPS-08	GCACGCCGGA	2	24	0	800–1500
9	OPS-09	AGGGGTCTTG	5	60	0	500–2000
10	OPS-10	TTATCTTGAC	2	24	0	450–1350
11	OPS-11	GGGTCTCGGT	4	48	0	300–2000
12	OPS-12	CGGTTGGTTA	3	36	0	250–600
13	OPS-13	AGAGCCGTCA	2	24	0	300–1000
14	OPS-14	CGGTATTATGC	5	60	0	550–1000
15	OPS-15	AGCCGTGGAA	6	72	0	100–2500
Total			49	588		
**Average**			**3.27**	**39.2**		

### RAPD analysis

All the reagents and chemicals necessary for PCR were obtained from Bangalore Genei Pvt., Ltd., Bangalore India. The PCR reaction was employed in a thermal cycler (Perkin Elmer, Gene Amp thermal cycler 2400) in an absolute volume of 25 μL, containing 25 ng template DNA, 10 picomoles of primer, 200 μM of each of the four dNTPs, 2.5 μL Taq buffer (10 mM Tris HCl pH 9.0, 50 mM KCl), 10 mM MgCl_2_, and 0.2 unit of Taq DNA polymerase. The mixed samples were treated with initial denaturation for 3 min at 94 °C, followed by 30 cycles of 1 min at 94 °C, 1 min at 36 °C, and an extension for 2 min at 72 °C with a final extension of 7 min at 72 °C. The eight μL of amplified PCR product was separated by gel electrophoresis on a 1.5% agarose gel stained with 0.5 μg μL^-1^ ethidium bromide. Finally, the separation of DNA was photographed with a gel documentation system (UVITEC, Cambridge, UK). The range of the separated PCR products was determined using a 100–3000 bp DNA ladder.

### Experimental design and statistical analysis

The full experiments were set up in a completely randomized block design (CRBD). There were 12 replicates per treatment, and all treatment was repeated three times. The data were evaluated with a one-way analysis of variance (ANOVA) using the SPSS version 25. The significant variances among the treatments were calculated using Duncan’s multiple range tests (DMRT). The treatment values were recorded as the mean ± SE. For RAPD analysis, only consistently reproducible and well-resolved bands ranging from 100 to 3000 bp in size were manually scored. The genetic associations were estimated by computing the Jaccard’s similarity coefficient (J) for pair-wise evaluations according to the portion of shared bands yielded by the RAPD markers. The similarity matrix was subjected to the cluster analysis of the unweighted pair group method with arithmetic averages (UPGMA). From these observations, a dendrogram was prepared using NTSYS-pc version 2.1 software [53].

## Results

### Shooting experiments

**Fig. 1:**
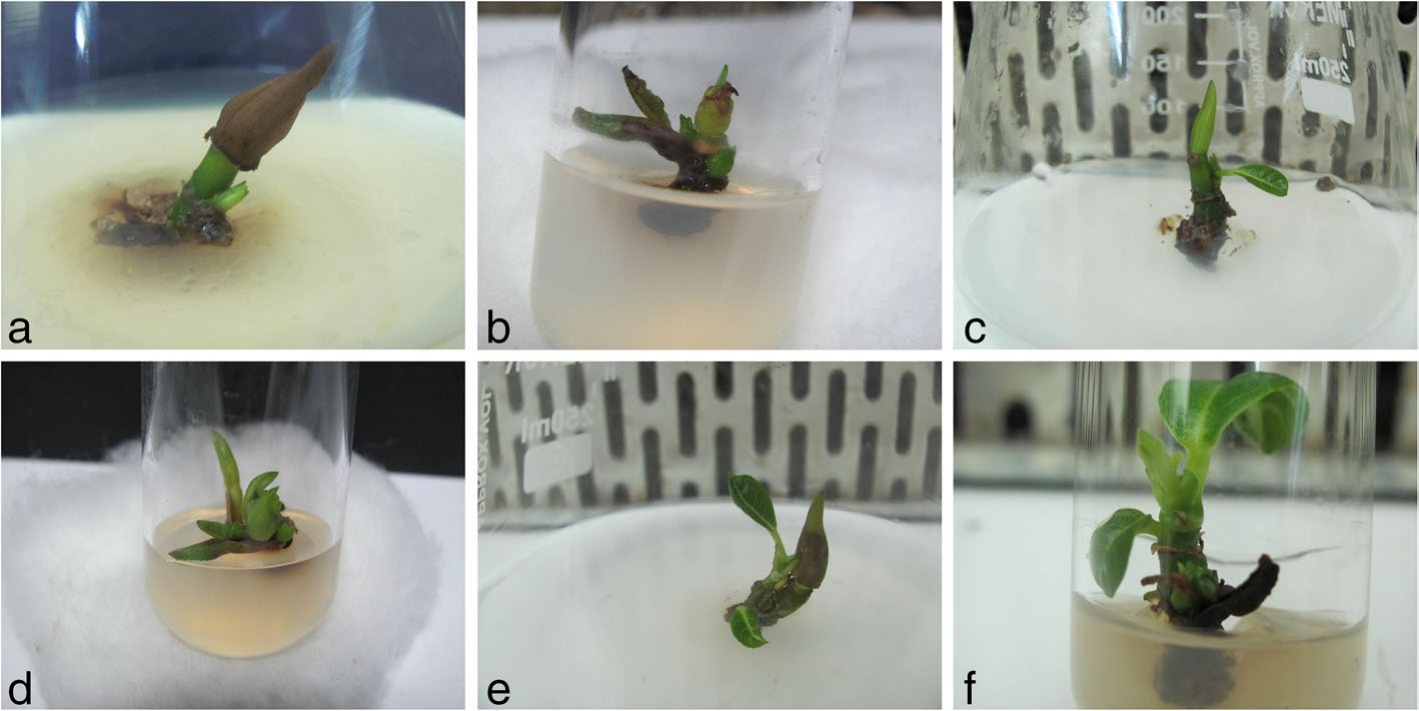
In vitro clonal propagation of *Artocarpus heterophyllus* L. **a** Initiation of the breakdown of shoot tip on full-strength Murashige and Skoog (MS) medium supplemented with 6-benzylaminopurine (BAP) at the concentration of 1.5 mg L^-1^, Indole-3-butyric acid (IBA) at 0.5 mg L^-1^, and α-naphthaleneacetic acid (NAA) at 0.1 mg L^-1^ in combination after 3–4 days of inoculation. **b** Initiation of the shoot (shown with black arrows) on MS medium formulated with BAP at the concentration of 1.5 mg L^-1^, IBA at 0.5 mg L^-1^, and NAA at 0.1 mg L^-1^ in combination after 12 days of inoculation. **c** A shoot having a leaf on MS medium formulated with BAP at the concentration of 1.5 mg L^-1^, IBA at 0.5 mg L^-1^, and NAA at 0.1 mg L^-1^ in combination after 22 days of inoculation. **d** Initiation of shoots on MS medium formulated with BAP at the concentration of 0.5 mg L^-1^ and 6-furfuryl-amino purine (Kn) at the concentration of 1.5 mg L^-1^ after 8 days of culture. **e** Shoots having multiple leaves on MS medium formulated with BAP at the concentration of 0.5 mg L^-1^ and Kn at the concentration of 1.5 mg L^-1^ after 22 days of culture. **f** Enlargement of shoot and leaf after the first subculture period on MS medium supplemented with 1.5 m gL^-1^ BAP, IBA at 0.5 mg L^-1^, and NAA at 0.1 mg L^-1^ in combination

The explant preparation and surface sterilization procedures described here yielded nearly 100% aseptic cultures in vitro (data not shown). No shoot formation occurred on the medium without any growth regulators even after 1 month of inoculation (Table 2), but when the explants were exposed to MS medium formulated with cytokinins and auxins at different concentrations and combinations or alone showed variation in the regeneration percentage and number of shoots formation. The concentration and type of cytokinins and auxins used significantly affected the percentage of regenerated shoots, shoot number, and shoot length. Different experiments of shooting are displayed in Table 2. The shoot tips started to break after 3–4 days of inoculation (Fig. 1a). From Table 2, it is found that BAP at the concentration of 1.5 mg L^-1^, IBA at 0.5 mg L^-1^, and NAA at 0.1 mg L^-1^ in combination yielded maximum shoot (Fig. 1b, c) induction rate (94.44%), highest average shoot length (4.02 cm), and the number of the shoot (Fig. 1d, e) per explant (4.78).

**Table 2:** The effects of different cytokinins and auxins on initiation and growth of shoots from shoot tips using the full strength of Murashige and Skoog medium of *Artocarpus heterophyllus* L.

Types and concentrations of plant growth regulators (mg L^**-1**^)				% of shoot response	Number of shoots per explant	Shoot length (cm)
BAP	Kn	NAA	IBA			
0	0	0	0	00±00^a^	00±00^a^	00±00^a^
0.5	0	0	0	22.22±0.07^bc^	1±00^b^	1.38±0.059^b^
1	0	0	0	33.33±0.079^bcde^	1.19±0.067^bc^	1.83±0.052^cdef^
1.5	0	0	0	44.44±0.084^cdefghi^	1.33±0.08^bcd^	1.93±0.051^defg^
2	0	0	0	38.89±0.082^bcdefg^	1.53±0.084^cd^	1.69±0.027^bcde^
0	0.5	0	0	16.67±0.063^ab^	1.28±0.076^bcd^	1.48±0.047^bc^
0	1	0	0	27.78±0.076^bcd^	1.31±0.078^bcd^	2.4±.475^hij^
0	1.5	0	0	36.11±0.081^bcdef^	1.33±0.08^bcd^	2.44±0.554^hij^
0	2	0	0	30.56±0.078^bcd^	1.44±0.084^cd^	1.5±0.035^bcd^
0.5	0.1	0	0	41.67±0.083^cdefgh^	2.22±0.07^gh^	1.92±0.04^defg^
1	0.1	0	0	58.33±0.083^efghijklm^	2.56±0.122^hij^	1.62±0.064^bcd^
1	0.2	0	0	69.44±0.078^ijklmn^	2.83±0.152^kl^	2.23±0.047^fghi^
1.5	0.1	0	0	58.33±0.083^efghijklm^	2.61±0.128^hjkl^	2.44±0.026^hij^
1.5	0.2	0	0	83.33±0.063^mn^	3.69±0.153^mno^	2.66±0.028^ijk^
1.5	0.5	0	0	77.78±0.07^lmn^	3.86±0.145^no^	3.26±0.038^mn^
2	0.5	0	0	69.44±0.078^ijklmn^	3.36±0.081^lm^	2.56±0.061^ijk^
0.1	0.5	0	0	36.11±0.081^bcdef^	1.36±0.081^bcd^	1.57±0.058^bcd^
0.1	1	0	0	52.78±0.084^defghijkl^	1.69±0.143^def^	2.05±0.06^efgh^
0.2	1	0	0	63.89±0.081^ghijklm^	2.31±0.111^ghi^	1.62±0.042^bcd^
0.1	1.5	0	0	47.22±0.084^defghijk^	1.67±0.16^de^	2.27±0.052^ghi^
0.2	1.5	0	0	75±0.073^klmn^	2.67±0.08^jkl^	2.76±0.051^jkl^
0.5	1.5	0	0	75±0.073^klmn^	4.42±0.184^p^	2.89±0.055^klm^
0.5	2	0	0	52.78±0.084^defghijkl^	2.86±0.133^kl^	2.27±0.085^ghi^
1	0	0.1	0.1	69.44±0.078^ijklmn^	2±0.138^efg^	3.13±0.029^lmn^
1.5	0	0.2	0.2	81.08±0.065^mn^	2.92±0.193^kl^	3.12±0.036^lmn^
1.5	0	0.5	0.1	80.56±0.066^mn^	3.75±0.14^no^	3.44±0.063^n^
**1.5**	**0**	**0.1**	**0.5**	**94.44±0.039**^**n**^	**4.78±0.145**^**q**^	**4.02±0.044**^**o**^
2	0	0.1	0.5	61.11±0.082^fghijklm^	3.33±0.211^lm^	3.43±0.041^n^
2	0	0.5	0.5	61.11±0.082^fghijklm^	3.78±0.16^no^	3.22±0.082^mn^
0	1	0.1	0.1	50±0.085d^efghijk^	2.31±0.104^ghi^	2.38±0.047^hij^
0	1.5	0.2	0.2	66.67±0.08^hijklm^	2.56±0.151^hij^	2.28±0.07^ghi^
0	1.5	0.5	0.1	72.22±0.076^jklmn^	2.06±0.138^fg^	3.26±0.06^mn^
0	1.5	0.1	0.5	80.56±0.067^mn^	3.94±0.138^no^	3.46±0.053^n^
0	2	0.1	0.5	72.22±0.076^jklmn^	3.64±0.208^mn^	3.17±0.132^lmn^
0	2	0.5	0.5	69.44±0.078^ijklmn^	3±0.138^lm^	2.91±0.053^klm^
1.5	0.5	0.1	0.1	75±0.073^klmn^	4.06±0.132^o^	3.46±0.049^n^

Here, *BAP* 6-benzylaminopurine, *Kn* 6-furfuryl-amino purine, *NAA* α-naphthaleneacetic acid, *IBA* Indole-3-butyric acid. Each mean value followed by the same letter does not differ significantly according to Duncan’s multiple range test (*p* ≤ 0.05). Marked in bold is the treatment that yielded the best result

### Effect of subculture on shoot multiplication

**Fig. 2:**
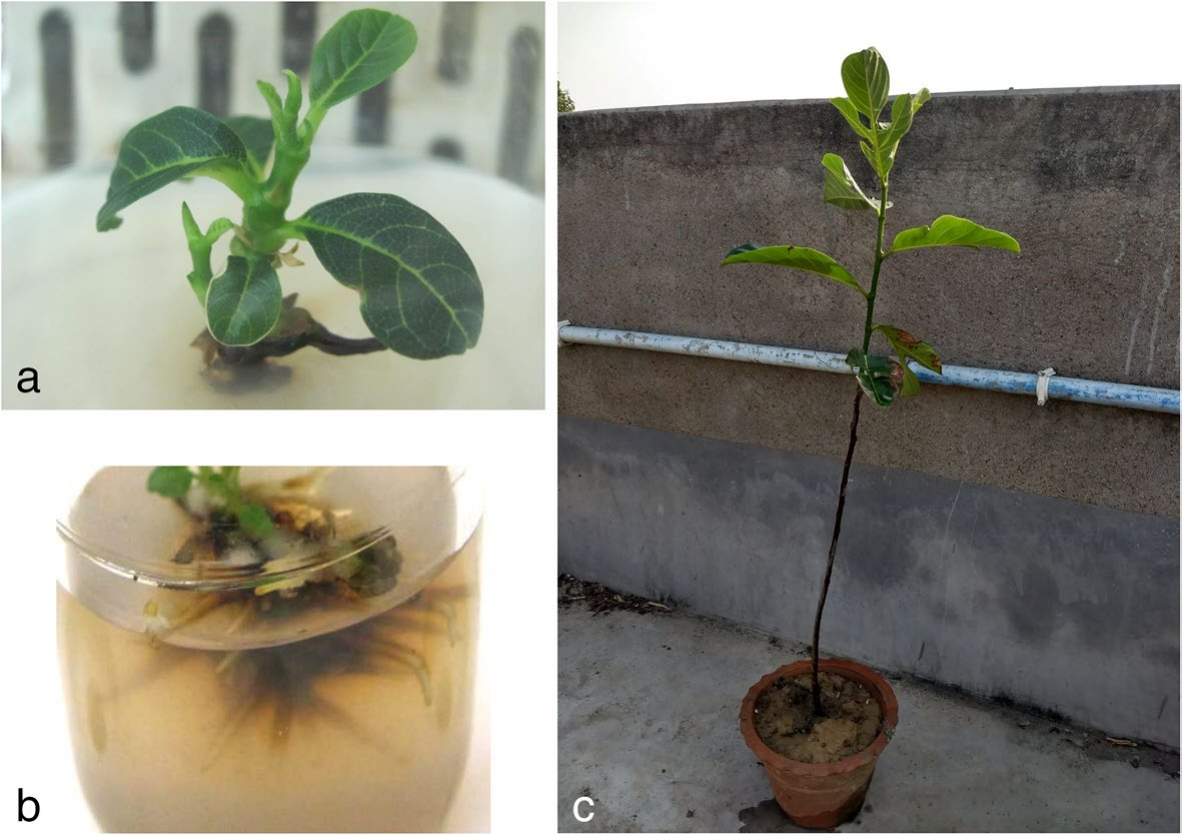
Micropropagation of *Artocarpus heterophyllus* L. **a** Enlargement and induction of new shoot and leaf after the second subculture period in Murashige and Skoog (MS) medium supplemented with 1.5 mg L^-1^ 6-benzylaminopurine (BAP), Indole-3-butyric acid (IBA) at 0.5 mg L^-1^, and NAA at 0.1 mg L^-1^ in combination. **b** Well development of roots in quarter strength of MS medium supplemented with 0.5 mg L^-1^ IBA and 0.1 mg L^-1^ BAP in combination. **c** Six-month-old secondary hardened plants in earthen pots

Induced shoots were multiplied by repeatedly subculturing the micro shoots on shoot multiplication medium (MS media supplemented with 1.5 mg L^-1^ BAP, IBA at 0.5 mg L^-1^, and NAA at 0.1 mg L^-1^ in combination) after 30 days of culture. The shoot regeneration capability was observed up to the sixth subculture passage (Table 3). From Table 3, it is found that the maximum number of shoots (5.92) with the largest shoot length (5.85 cm) was found in the third subculturing period (Figs. 1f and 2a) and reduced thereafter.

**Table 3:** Effect of repeated subcultures of microshoots on full-strength Murashige and Skoog medium supplemented with 1.5 mg L^-1^ 6-benzylaminopurine, Indole-3-butyric acid at 0.5 mg L^-1^, and α-naphthaleneacetic acid at 0.1 mg L^-1^ on shoot proliferation of *Artocarpus heterophyllus* L.

Subculture	Number of shoots/explant	Shoot length (cm)
First	4.67±0.08^a^	4.32±0.03^a^
Second	4.97±0.13^ab^	4.92±0.04^b^
**Third**	**5.92±0.14**^**d**^	**5.85±0.04**^**d**^
Fourth	5.39±0.16^c^	5.18±0.05^c^
Fifth	5.31±0.11^bc^	4.9±0.03^b^
Sixth	5.19±0.09^bc^	5.1±0.03^c^

Each value represents the mean ± SE of three repeated experiments. Each mean value followed by the same letter does not differ significantly according to Duncan’s multiple range test (*p* ≤ 0.05). Marked in bold is the treatment that yielded the best result

### Effect of different plant growth regulators on in vitro rooting of microshoots

The formation of roots from micro shoots is essential to facilitate their establishment in soil. Auxins motivate the happening of adventitious roots in many plant species. Therefore, in this study, the effects of auxins (IBA and NAA) on the rooting of regenerated shoots were examined (Table 4). Rooting experiments were performed after shoots from the third subculture passage were transferred to different strengths of MS media like full-strength MS medium, half-strength (½) MS medium, and quarter strength (¼) MS medium supplemented with 0.1 mg L^-1^ IBA and without growth regulator in all media. The results of this experiment displayed that there was no formation of roots without a plant growth regulator. Apart from this, the full-strength MS medium with 0.1 mg L^-1^ IBA also showed no root formation even after 1 month of inoculation. However, among the different strengths of MS media screened for rooting (Table 4), the ¼ MS media with 0.1 mg L^-1^ IBA was found to be best for rooting experiments including the percentage of root response (27.78%), number of roots per cutting (2.42), and for average root length (0.69 ± 0.02 cm). From Table 4, it is shown that the combination of 0.1 mg L^-1^ NAA, 0.5 mg L^-1^ IBA, and 0.1 mg L^-1^ BAP exhibited a superior level of root response (94.44%), the maximum number of roots per shoot (8.44), and the highest average length of root (3.78 cm).

**Table 4:** The effects of various auxins and cytokinins on the initiation and growth of in vitro roots on different strengths of Murashige and Skoog medium of induced micro shoots of *Artocarpus heterophyllus* L.

Media	Types and concentrations of plant growth regulators (mg L^**-1**^)				% of root response	Number of roots/cutting	Root length (cm)
	NAA	IBA	BAP	Kn			
MS	0	0	0	0	00±00^a^	00±00^a^	00±00^a^
½ MS	0	0	0	0	00±00^a^	00±00^a^	00±00^a^
¼ MS	0	0	0	0	00±00^a^	00±00^a^	00±00^a^
MS	0	0.1	0	0	00±00^a^	00±00^a^	00±00^a^
½ MS	0	0.1	0	0	13.89±0.058^ab^	1.33±0.08^b^	0.28±0.034^b^
¼ MS	0	0.1	0	0	27.78±0.076^bcd^	2.42±0.122^cd^	0.69±0.02^d^
¼ MS	0.15	0	0	0	22.22±0.07^abc^	2.33±0.08^c^	0.44±0.015^c^
¼ MS	0.2	0	0	0	22.22±0.07^abc^	2.64±0.15^cde^	0.47±0.022^c^
¼ MS	0.5	0	0	0	38.89±0.082^cdef^	3.42±0.212^ghi^	0.76±0.022^d^
¼ MS	0	0.15	0	0	33.33±0.08b^cde^	2.47±0.101^cd^	0.84±0.008^d^
¼ MS	0	0.2	0	0	63.89±0.081^ghijk^	3.08±0.237^efghi^	1.140.038^e^
¼ MS	0	0.5	0	0	61.11±0.082^fghijk^	3.11±0.137^efghi^	1.3±0.041^efg^
¼ MS	0.5	0.1	0	0	58.33±0.083^fghij^	3.5±0.14^ghi^	1.55±0.052^h^
¼ MS	0.1	0.5	0	0	66.67±0.08^ghijk^	4.92±0.146^k^	2.21±0.058^jkl^
¼ MS	0.1	0	0.05	0	22.22±0.07^abc^	1.81±0.096^b^	1.21±0.041^ef^
¼ MS	0.2	0	0.05	0	33.33±0.08b^cde^	1.62±0.081^b^	1.52±0.056^h^
¼ MS	0.5	0	0.1	0	58.33±0.083^fghij^	2.75±0.092^cdef^	2.33±0.049^kl^
¼ MS	0	0.1	0.05	0	33.33±0.08^bcde^	5.42±0.25^klm^	1.36±0.042^fg^
¼ MS	0	0.2	0.05	0	47.22±0.084^defgh^	4.94±0.36^k^	2.67±0.05^m^
¼ MS	0	0.5	0.1	0	66.67±0.08^ghijk^	6±0.21^n^	3.01±0.049^n^
¼ MS	0.1	0	0.05	0.05	44.44±0.084^cdefg^	3.31±0.21^fghi^	1.17±0.062^e^
¼ MS	0.2	0	0.05	0.05	55.56±0.084^efghi^	3.64±0.29^i^	1.27±0.043^ef^
¼ MS	0.5	0	0.1	0.1	55.56±0.084^efghi^	3±0.15^defgh^	2.07±0.068^j^
¼ MS	0	0.1	0.05	0.05	75±0.073^ijkl^	3.6±0.17^i^	1.26±0.041^ef^
¼ MS	0	0.2	0.05	0.05	83.33±0.063^jkl^	4.17±0.20^j^	1.77±0.068^i^
¼ MS	0	0.5	0.1	0.1	77.78±0.07^ijkl^	5.56±0.2^lmn^	2.36±0.052^l^
¼ MS	0.1	0	0	0.05	63.89±0.081^ghijk^	2.86±0.16^cdefg^	1.44±0.03^gh^
¼ MS	0.2	0	0	0.05	69.44±0.078^ghijkl^	4.22±0.23^j^	1.84±0.092^i^
¼ MS	0.5	0	0	0.1	69.44±0.078^ghijkl^	3.5±0.11h^i^	2.35±0.041^kl^
¼ MS	0	0.1	0	0.05	72.22±0.076^hijkl^	2.56±0.09^cde^	1.79±0.066^i^
¼ MS	0	0.2	0	0.05	72.22±0.076^hijkl^	5.2±0.23^kl^	2.19±0.094^jk^
¼ MS	0	0.5	0	0.1	72.22±0.076^hijkl^	5.14±0.11^kl^	2.6±0.07^m^
¼ MS	0.1	0.1	0.05	0.05	75±0.073^ijkl^	5.03±0.14^kl^	2.66±0.088^m^
¼ MS	0.2	0.2	0.05	0.05	72.22±0.076^hijkl^	5.95±0.32^mn^	3.17±0.042^o^
¼ MS	0.5	0.5	0.1	0.1	69.44±0.078^ghijkl^	6.08±0.22^n^	3.55±0.046^p^
¼ MS	0.5	0.1	0.1	0	75±0.073^ijkl^	7.17±0.23^o^	3.57±0.044^p^
**¼ MS**	**0.1**	**0.5**	**0.1**	**0**	**94.44±0.039**^**l**^	**8.44±0.13**^**p**^	**3.78±0.068**^**q**^
¼ MS	0.5	0.1	0	0.1	83.33±0.063^jkl^	8.03±0.18^p^	3.48±0.088^p^
¼ MS	0.1	0.5	0	0.1	86.11±0.058^kl^	7.33±0.21^o^	3.41±0.057^p^

Here, *NAA* α-naphthaleneacetic acid, *IBA* Indole-3-butyric acid, *BAP* 6-benzylaminopurine, *Kn* 6-furfuryl-amino purine. Each value represents the mean ± SE of three repeated experiments. Each mean value followed by the same letter does not differ significantly according to Duncan’s multiple range test (*p* ≤ 0.05). Marked in bold is the treatment that yielded the best result

### Acclimatization and field establishment

The fruitful acclimatization of micropropagated plants and their succeeding transfer to the field is a fundamental step in the commercial exploitation of in vitro technology. In this present study, the whole plantlet regeneration was obtained within 200 days of culture. Primarily the plants were acclimatized to the ex vitro controlled environment in plastic cups having a sterilized mixture of garden soil and vermiculite mixture (1:1, w/w) for primary hardening. After 4 weeks of primary hardening, the mean survival percentages of the potted plants were 88.89% (Table 5). After 1 month of primary hardening, randomly selected thirty nine successfully primary hardened plants were shifted to earthen pots containing a vermiculite-soil mixture (2:1; w/w) under greenhouse conditions for the secondary hardening process. About 90.60% of plants subjected to secondary hardening survived after 60 days and could be grown in pots kept in a greenhouse or under open conditions (Fig. 2c).

**Table 5:** Acclimatization of *Artocarpus heterophyllus* L.

Experiment	Primary hardening		Secondary hardening	
	Plant transferred	% of survival after 3 weeks	Plant transferred	% of survival after 3 weeks
Experiment 1	33	87.88	39	87.18
Experiment 2	33	84.85	39	89.74
Experiment 3	33	93.94	39	94.87
**Mean**		**88.89 ± 1.7**		**90.60 ± 2.3**

### RAPD analysis

**Fig. 3:**
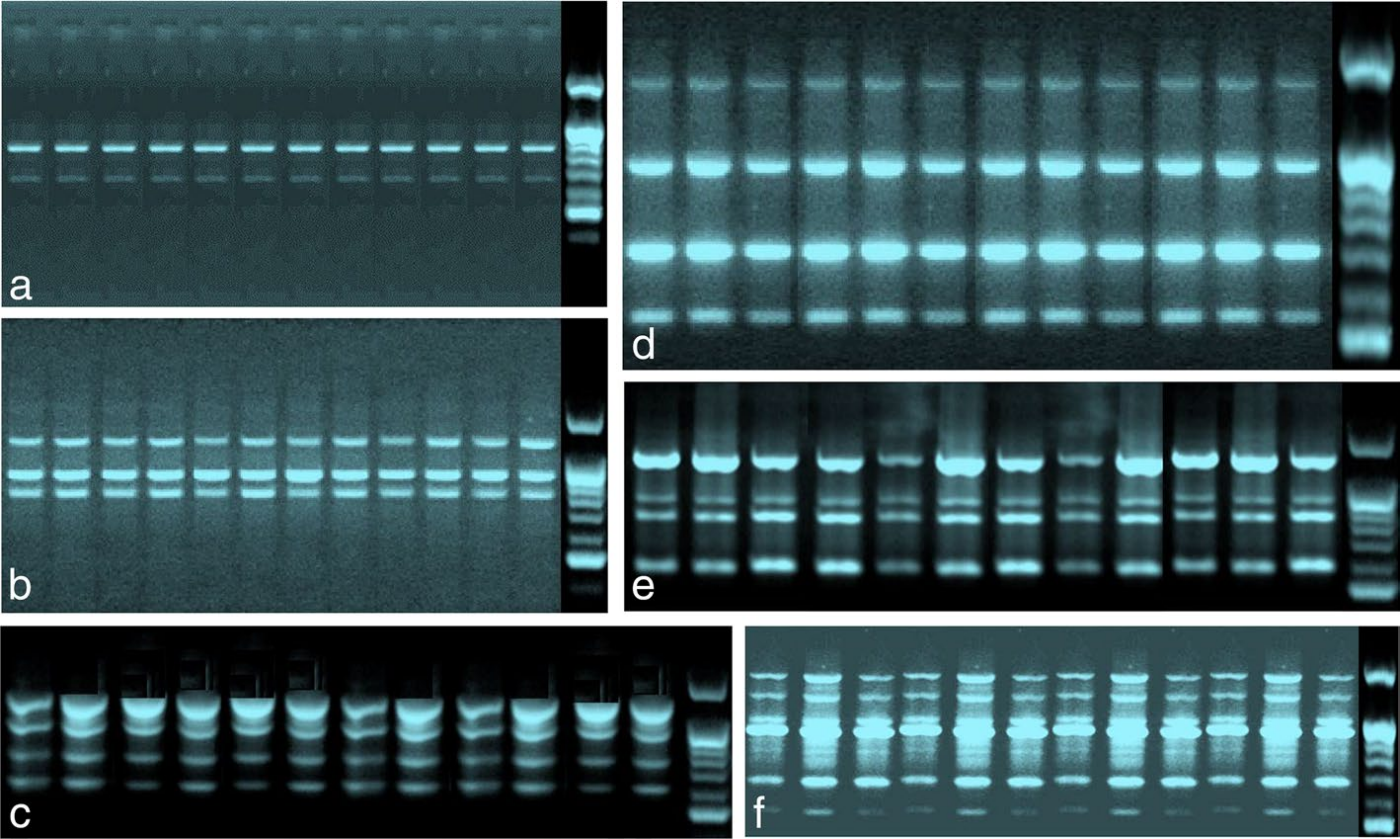
Polymerase chain reaction (PCR) amplification products were obtained with random amplified polymorphic DNA (RAPD) primers. M for the mother plant, 1–11 represent the micropropagated hardened plants, and MA for the DNA ladder. **a** PCR amplification with RAPD primer OPS-01. **b** PCR with RAPD primer OPS-02. **c** PCR with RAPD primer OPS-05. **d** PCR with OPS-07. **e** PCR with RAPD primer OPS-11. **f** PCR with RAPD primer OPS-15

It is of capital importance to assess the genetic stability of the regenerated hardened plant prior to determining the success of an in vitro protocol. In this context, fingerprinting profiles of the hardened plants and the donor plant were done by RAPD markers to check whether the plantlets were genetically stable or transformed. DNA from randomly selected 11 micropropagated hardened plants of jackfruit trees was compared with the DNA of the mother plant to confirm the genetic stability. In the present investigation, a total of 27 RAPD primers were executed for initial screening, among them, only 15 primers displayed clear and reproducible loci. The total number of scorable loci for all RAPD primers varied from 2 to 6 (Table 1). The 15 primers employed in this analysis generated 49 scorable loci with an average of 3.27 fragments per primer. Every primer yielded a unique set of amplification products ranging in size from 100 to 2400 bp. A total of 588 bands (number of samples analyzed × number of scorable loci) were yielded by 15 RAPD markers, and all bands were found to be monomorphic. No polymorphism was detected during the RAPD profiling of the donor mother plant with in vitro raised micropropagated hardened plants (Fig. 3). The similarity matrix revealed that the pair-wise assessment of the hardened plants and the mother plant was 1, demonstrating 100% resemblance. This established the true-to-type nature of the tissue culture grew regenerants and certified that the hardened plants were free from any somaclonal variation. Through PCR-based RAPD analysis, it was established that the uniformity of the regenerated plants was sustained, indicating high genetic stability among the clones.

## Discussions

### Shooting experiments

The type of explant applied in micropropagation is a crucial step in tissue culture studies due to several factors, like juvenility vs. maturity, inherent slow-growing pattern, exogenous and endogenous microbial infection, the occurrence of phenolic compounds, long complex life cycles, and genetic differences [25]. Studies by Amany et al. [3] and Harb et al. [25] showed that the shoot tip was more efficient in shoot multiplication studies rather than inter nodal segments with this species. Moreover, Harb et al. [25] reported that shoot tips had more survival rate percentage and less phenolic contamination of explants than nodal segments in all seasons. A similar result was also reported by several authors all over the world with this species [2, 7, 38]. Table 2 shows that BAP and Kn both singly or in combination encouraged the production of axillary shoots in various concentrations. This may be due to cytokinin is known as one of the vital hormones for plant growth and development that is known to encourage cell division and differentiation of various plant parts. This type of growth regulator also participated in various physiological activities like activation of RNA, protein synthesis, and various enzyme activities [21]. Table 2 also showed that BAP alone has much more effects on the percentage of shoot response than Kn alone. The superiority of BAP over Kn was also documented by various authors with this species [4, 5, 25]. BAP causes reinvigoration of mature tissues and causes bud induction which is a prerequisite for cloning mature trees [73]. Furthermore, it is reported that BAP can surpass dominance which can affect the breakdown of dormancy of lateral buds and stimulate shoot formation. The addition of a lower concentration of auxins along with higher concentrations of cytokinin has tremendous effects on shoot multiplication. In this study, the addition of a lower concentration of auxins (IBA and NAA) along with a higher concentration of cytokinin accelerated the shoot multiplication investigation. This type of result may agree with other authors on this species [2, 3].

### Effect of subculture on shoot multiplication

For large-scale commercial multiplication, a cost-effective process is very necessary. Subculturing of microshoots significantly improved multiple-shoot induction and direct organogenesis. Earlier in vitro studies of this species mostly focus on different medium compositions other than propagation medium for subculturing, to facilitate the multiplication of in vitro shoots [3, 4, 8, 25, 33]. However, the present investigation employed the same media composition for the establishment of shoot induction and their subsequent multiplication. This approach does not need any addition of extra compound or depletion of medium component for shoot multiplication. In this respect, the present protocol was found to be easy, fast, reliable, and attractive for significant shoot bud induction and multiplication. Moreover, it was reported that the application of different medium compositions rather than propagation medium makes the in vitro process long, cumbersome, and inefficient [9]. To support the observations of the present study, a similar type of results was also coined by Patel et al. [47] and Shekhawat et al. [59] for *Caralluma edulis* and *Morinda citrifolia* species, respectively. They reported the enhancement of shoot numbers per explant up to the third subculture stage. In connection with this, it was documented that shoot multiplication events are enormously altered by the number and duration of subcultures. Sequential subculture events in cytokinin-enriched media improve the shoot multiplication efficiency in many plant species even though the cultures may degenerate [28, 69]. This happened may be due to additional time in a culture that copes with repeated subculturing can increase multiplication efficiency, which can further facilitate incomplete development [20]. Moreover, the horizontal position of explants may improve shoot number which was reported earlier in the micropropagation of a woody tree species. The shoot enhancement problems that took place after the third subculture may be due to the enrichment of cytokinin in media. Other reasons might also be associated such as the higher level of moisture in the culture and/or the existence of ethylene, and the time taken to transfer cultures. In this circumstance, Torregrosa and Bouquet [69] stated that more than three subculture phases led to the formation of abnormal shoots and a sharp reduction in the multiplication capacity of the axillary bud explants.

### Effect of different plant growth regulators on in vitro rooting of microshoots

The strength of the MS medium is a vital feature in manipulating the rooting efficiency. Previous reports of in vitro rooting of this species were mostly found to be best on ½-strength MS media [7, 8, 25, 38]. The present study too exhibited rooting on half-strength MS media. However, the superior level of in vitro rooting was achieved with ¼-strength MS media. This type of result was coined by Chaturvedi et al. [15] with *Azadirachta indica*. Among the two different types of auxins examined, the IBA had a more pronounced effect on in vitro rooting than NAA. This finding may correlate with the findings of other authors on this species [4, 5, 8, 25]. The possible reason for IBA’s better efficiency could be because IBA is more stable at room temperature and also upon autoclaving [45]. A few reports like Amin and Jaiswal [4] and Ara et al. [5] showed that the micro cuttings produced roots in vitro in the absence of auxins. Unlike this observation, the present study displayed that there was no formation of roots without any growth regulators in all strengths of the MS media tested. Moreover, there was no stimulation of the root on the full strength of MS media complemented with 0.1 mg L^-1^ IBA. Repeated subculture into fresh medium gradually enhanced rooting frequency because repeated subculturing may alter the physiological state and progressively rejuvenate the shoot, which in turn encourages better rooting.

### RAPD analysis

In long-lasting tree species, where adverse effects are expressed in the field only after years, this may be economically unsuccessful. Therefore, true-to-type propagation of the designated woody species is preferred. Clonal fidelity and genetic stability study of tissue culture material for several utilities including forestry may help to cut the extensive field-testing trials [26, 55]. To assess this, the most reliable approaches are the molecular marker technology that identifies the change depending on the defined regions of DNA or DNA polymorphisms [36]. This was most probably due to somaclonal variations which may appear because of cell cycle disorders caused by exogenously functional growth regulators particularly 2,4-dichlorophenoxyacetic acid (2,4-D) which is used as auxin and is known to be a mutagen [26, 48, 55, 64], changes in chromosome structure (duplications, translocations) or in chromosome number (leading to polyploidy) [17], DNA damage and mutation, change in DNA methylation patterns [49], gathering of mutations (activation of transposons) over a period of time and increased mutation rate per cell-generation over time [52], and modification of cell’s capability to repair mutated and damaged DNA [39]. Furthermore, it is documented that tissue culture standardizes a physiological strain which is characterized by the interference of normal developmental panels that can ultimately lead to several types of deviations at the nucleotide sequence level [13]. Being a complex developing procedure, somatic embryogenesis may be associated with the variation-induced fault in any desired characters and involves the combined action of many genes [26]. Several influences like the number of subcultures, genotype, time of culture, explant source, the genetic mosaicism and level of ploidy, media composition, and the most important pattern of regeneration, i.e., involving callus phase or direct organogenesis can promote in vitro mutability [1, 36, 55, 56, 61, 72]. Normally, to evaluate genetic stability, various molecular techniques have been employed, but probably, the most frequently used and conclusive are RAPD, ISSR (Inter-simple sequence repeat), and AFLP (amplified fragment length polymorphism) due to their robustness and huge screening of the genome [16]. Literature studies revealed that RAPD is very much attractive over them in the detection of genetic variability as for its technical simplicity, requires only small amounts of DNA, relatively low cost, and is quick to perform [34, 55, 70]. Furthermore, an additional benefit is that this method can be applied to diverse species without prior genetic knowledge. Due to this reason, this technique is widely applied for evaluating clonal fidelity and genetic stability of micropropagated plants in a number of genera. RAPD endorsed the absence of somaclonal variation in micropropagated plant material reported earlier for several tree woody species like *Acacia auriculiformis* [71], *Ficus religiosa* [63], and a well-known medicinal plant *Azadirachta indica* [6], etc. Likewise, some researchers have also noted polymorphism in micropropagated plants through RAPD markers like *Beta vulgaris* [43], *Robinia pseudoacacia* [11], *Cineraria maritime* [67], etc. The RAPD markers can generate a higher number of bands, thereby constructing an additional representative sample of the genome than is possible with allozyme [58]. Moreover, RAPD provides numerous benefits over restriction fragment length polymorphism (RFLP) in various ways like no previous cloning of DNA sequences is obligatory, requires very little amount of plant tissue, and is associated with a rapid DNA extraction procedure [26, 51]. RAPD marker exposes DNA polymorphism as practices primers of random sequences that examine for complementarities in the genome and variances in the amplification patterns. This is proposed that DNA bands probably characterize repetitive sequences [23, 40]. The variation and or polymorphism in repetitive DNA sequences has normally been detected during plant tissue culture studies [57, 65]. The tissue culture stress may give rise to deviations at privileged sites, like repetitive DNA, thereby stimulating transposons. With respect to decreasing the amount of somaclonal deviation to a commercially adequate level, this is suggested that multiplication and subcultures should be restricted to 1000 plantlets per explant, and micropropagated hardened plants should be screened in the nursery for initial recognition of variations [36]. Regeneration of plants from organized meristems is allowed to be the most trustworthy technique of micropropagation, as they are noted to be resistant to genetic deviation induced by in vitro techniques [60]. The plantlets that were achieved from current research were obtained from explants with a view to escaping long periods of unorganized mass (callus) development. Apart from this, this study also avoided the use of 2,4-D and substituted it with NAA and IBA which are considered as less mutagenic than 2,4-D [26]. In addition to this, this study exhibited the maximum shoot multiplication within the third subculture period which can be considered a “safe level” of multiplication. There are numerous kinds of DNA deviation that RAPD primers are unable to identify, including repetitions of chromosomes or genes and happening of occasional point mutations [26]. These deviations, however, are very tough to identify by any recent approach of DNA assay in related sceneries. These outcomes accomplished that no somaclonal variant could be noticed according to this protocol, using the RAPD fingerprinting technique. Like this study, the dendrogram based 100% true-to-type nature of clones to their donor plant was also reported earlier by Sreedhar et al. [66], Bhatia et al. [10], and Singh et al. [62] for *Vanilla planifolia*, *Gerbera jamesonii*, and *Dendrocalamus asper* species, respectively.

## Conclusion

This is the first report of the genetically stable micropropagation protocol of *Artocarpus heterophyllus* (Jackfruit tree) using shoot tip as an explant. This study exposed that multiple shoot bud stimulation and regeneration of *A. heterophyllus* were controlled by suitable cytokinin and auxin concentrations and a combination of plant growth regulators. The process of in vitro rooting coupled with immediate hardening reduced the time of the micropropagation protocol and made the protocol more economical. Due to the endorsement of genetic sustainability, this technique seems to be steady and supports that micropropagated plantlets are true to the type of the donor mother plant. This study set up an effective and reliable micropropagation protocol for in vitro regeneration of *A. heterophyllus* to guarantee large-scale propagation towards a sustainable supply of plant resources to the various industries or conservation of elite germplasm.

## Data Availability

All data generated or analyzed during this study are included in this published article [and its supplementary information files].

## Abbreviations

MS: Murashige and Skoog
Kn: 6-Furfuryl-amino purine
BAP: 6-Benzylaminopurine
NAA: α-Naphthaleneacetic acid
IBA: Indole-3-butyric acid
